# Patient and public involvement in Paediatric Intensive Care research: considerations, challenges and facilitating factors

**DOI:** 10.1186/s40900-016-0046-7

**Published:** 2016-11-07

**Authors:** J. C. Menzies, K. P. Morris, H. P. Duncan, J. F. Marriott

**Affiliations:** 1 Paediatric Intensive Care Unit, Birmingham Children’s Hospital, Steelhouse Lane, Birmingham, B4 6NH UK; 2 Institute of Clinical Sciences, College of Medical and Dental Sciences, Medical School Building, University of Birmingham, Edgbaston, Birmingham B15 2TT UK

**Keywords:** Consumer involvement, Consultation, Research design, Paediatric intensive care, Patient and public involvement, PPI

## Abstract

**Plain English summary:**

Paediatric Intensive Care (PIC) provides care to extremely ill children. Research in this area can be difficult because children are often too sick to discuss being involved in a study and parents are too upset about their child to think about taking part. This makes it even more important that research is well designed. We conducted a review of the literature about involving patients and the public (PPI) in PIC research. We wanted to know what PPI has taken place, who had been consulted and how this was undertaken. We reviewed the titles and abstracts of 4717 papers but found only 4 relevant papers. Three of the papers had consulted with parents of children who had been on PIC but only one study had spoken directly to a child themselves. The studies had used a number of different methods to invite people to take part but there did not appear to be one solution. All of the studies thought PPI was good for the development of their research but none of them had tried to measure what had changed as a result. There are difficulties associated with carrying out PPI in the PIC setting. Researchers need to share more of their experiences, positive and negative, so we can try to identify the best ways of carrying out PPI in PIC studies. This will help ensure that research studies are designed which address the needs and concerns of children and their parents.

**Abstract:**

**Introduction**

Involving the public in health care research is reported to enhance the quality, appropriateness, acceptability and relevance to patients and the public (INVOLVE, Briefing notes for researchers, 2012; Staniszewska et al., Int J Technol Assess Health Care 274:391-9, 2011). Conducting research with children and young people is regarded as challenging and this makes it even more important that the research is well designed and understands the perspective of the child and family. We conducted a narrative literature review of the Patient and Public Involvement (PPI) literature, in the context of Paediatric Intensive Care (PIC). Our aims were to identify what PPI activity has taken place, with whom researchers engaged and what outcomes they reported.

**Method**

Electronic databases Medline, CINAHL and Embase (January 2000- June 2016) were searched using the search terms patient and public involvement and consultation. Participants were defined as child, parent, paediatric or pediatric and the context as intensive or critical care. Papers were excluded where activity reflected ‘participants’ as research subjects. Included papers were reviewed using the GRIPP checklist to appraise the quality of reporting.

**Results**

The search strategy identified 4717 abstracts. Seventeen papers were reviewed in full and four papers were included, all of which are case studies, describing a consultation approach. None of the papers described PPI as a multi-stage process. Only one study engaged with a former PIC patient and the majority of those consulted did not have any PIC experience. Activity was reported as being of benefit but there was no measurement of the impact of PPI.

**Conclusion**

There are numerous challenges associated with the conduct of research in PIC. It is therefore essential that the perspective of children, young people and their parents have been considered in the design of trials. However, there are few published accounts of PPI within the PIC context and the accounts that exist highlight issues about who to approach and when, and a lack of clarity about the best ways to engage with them. Research Ethics Committees and funding bodies expect to see evidence of PPI in research applications and we need to develop our understanding of what contributes towards successful PPI in this context.

Please see Glossary in Table 1 for abbreviations and definitions.

**Table 1:** Glossary

Research Ethics Committees (REC): review applications for research in the NHS and other specific situations and give an opinion about the proposed participant involvement and whether the research is ethical [7].	
Patient and Public Involvement (PPI): research being carried out ‘with’ members of the public, rather than ‘to’ or ‘for’ them [26].	
Paediatric Intensive Care (PIC): are units specifically providing care for critically ill children from pre term neonates to adolescents [43].	
National Institute for Health Research (NIHR): is an organisation funded through the Department of Health to improve the health of the nation through research [83].	
NIHR Young Person Advisory Group (YPAG): There are six UK YPAG groups who influence the development and conduct of clinical research through the input of children and young people [60].	
Involvement is used to describe service user active involvement in PPI activity (research design and process) [26].	
Participation is used to reflect members of the public taking part in a research study as subjects [26].	
We have used the term ‘child’ or ‘children’ throughout the article to represent children and young people.	

## Background

Those who are affected by research have a right to have a say in what and how research is undertaken [1, 2]. Involving the public in health care research is reported to enhance the quality and appropriateness of research, develop user-focused research objectives and information, develop questionnaires and interview schedules and lead to more appropriate recruitment strategies [3]. Patient and Public Involvement (PPI) activity is increasing significantly, reflecting the emphasis of national policy to embed PPI in research [4, 5]. Clinical or health researchers are expected to involve the public in their research and there should be evidence of this within Research Ethics Committee (REC) applications [6, 7] and within funding applications [8].

### PPI in paediatric research

The United Nations Convention of the Rights of the Child [9] gives children a right to be involved in decisions that affect their lives and for their views to be listened to. Involving patients in their care and treatment improves their health outcomes, boosts their satisfaction with services and their adherence to treatment [10]. The principle of ‘no decision about me, without me’ is central to this and applies as much to children and young people as any other patient group [11]. In a clinical or health research setting this has resulted in a shift towards engaging children directly in research, rather than relying on parents or carers to represent their children [12–14]. This approach to research conduct, including PPI activities, recognises that children have knowledge that is separate from their parents and carers knowledge and that this knowledge is worthy of consideration [15]. However in order to engage with children researchers will have to negotiate with parents and carers as ‘gatekeepers’ [16, 17]. Parents may also want to become involved in PPI in their own right but care must be taken to keep the two groups separate, respecting children and young people’s right to privacy and the likelihood that the inherent power differential between adults and children would make collaboration difficult [15]. Involvement of children and young people and parents should enable researchers to design studies that are more acceptable and relevant to service users. However despite this a recent survey of National Institute Health Research (NIHR) funded trials (2006–2010) found declining rates of PPI within paediatric trials [18]. This is of concern because conducting clinical trials in children is generally regarded as more challenging than those in adults and omitting PPI could affect recruitment, appropriate design and prioritised outcomes [19–21]. Researchers must make efforts to understand the perspective of service users through direct engagement in PPI activities [22]. Research Ethics Committees (REC) require researchers to demonstrate evidence of PPI activity with children and parents to ensure that a study design is appropriate; risks and burdens have been minimised and information materials are comprehensible to their target audience. Where the question relates to what a specific population of children or young people might feel, for example in the case of a serious chronic condition, they advise that more specialised input is appropriate [23]. This is challenging in an area such as Paediatric Intensive Care where there is huge diversity amongst service users.

As researchers’ designing and conducting studies in the Paediatric Intensive Care (PIC) setting we experienced a number of challenges to conducting PPI. We therefore conducted a narrative literature search surrounding Patient and Public Involvement (PPI) and Paediatric Intensive Care (PIC) to explore the existing evidence base. A systematic review was not felt to be appropriate because although the approach allows for a less biased appraisal of the evidence [24], the emphasis is often on a focused clinical question. PPI activity is often poorly defined, the method of conduct can vary widely according to the specific study aims and there is often limited evidence provided to demonstrate the impact of interventions [25]. We therefore felt that a narrative review would be the most constructive approach to scope the broad type of activity that has taken place, identify with whom researchers have engaged, methods of engagement and identify recommendations for future researchers.

## Method

### Inclusion criteria

Electronic databases searched comprised Medline, CINAHL and Embase for the period January 2000- June 2016. Searches were limited to those written in the English language, where involvement was referred to within the title or abstract and where there was an abstract available. There is no Medical Subject Heading (MeSH) for PPI so a combination of terms was used including involvement, consultation and patient and public involvement. Participants were defined as Paediatric, Pediatric, Child and Parent and the setting defined by Intensive or Critical Care. Inclusion criteria was the published account of ‘service users’ defined as children and their families who had been admitted to a Paediatric Intensive Care Unit or who were naïve to the setting but had agreed to be consulted about research in this specific setting. Papers were excluded if the consultation was with health services providers, rather than service users, or the activity reflected involvement in an educational intervention or service development. Following definitions by INVOLVE [26] we excluded papers where ‘participants’ reflected members of the public taking part in a study as research subjects, indicated by Research Ethics Committee approval and an informed consent process. Recognising that PPI can refer to a range of activities, eligibility criteria were broad to capture any activity which described involving children and their families in research design and conduct. Included papers were reviewed using the GRIPP (Guidance for Reporting Involvement of Patients and Public) checklist [27] to appraise the quality of PPI reporting. We also searched for any additional publications and guidance documents from a number of sources including EUPATI (European Patients’ Academy on Therapeutic Innovation), NIHR, INVOLVE as well as reference lists from published work.

## Results

A total of 4717 titles and abstracts of studies were screened by one reviewer (JCM) to identify potentially relevant publications. Please see Table 2 for characteristics of included studies. Where the abstract was unclear the full text was obtained. A total of 400 titles and abstracts were screened by a second reviewer (KPM) to check inclusion and exclusion criteria. Where there was disagreement a third reviewer was consulted (JFM). Of 4717 studies only 17 papers were reviewed in full as they potentially featured public involvement with service users associated with a paediatric critical care setting. These were reviewed in full by two reviewers (JCM & KPM). Thirteen papers were excluded because they did not meet the study’s inclusion criteria. Reasons for exclusion included- 5: review papers (2 in Neonatal Intensive Care Unit (NICU) setting, 1 review of palliative care discharge from PIC, 1: fundraising for research and 1 guidance document on best practice in deferred consent in paediatric research), 4: deemed research rather than PPI (publication was called ‘study’ or ‘research’ by authors, approved by an Institutional Review Board or REC and participants consented to participate), 2: non PIC setting (adult critical care setting), 1: conducted with health professionals (nurses). Four papers were included, all of which are case studies; 3 are reported in abstract form only. Searching the grey literature found a number of guidance documents produced by Royal College of Paediatrics and Child Health (RCPCH) [28], Nuffield Council on Bioethics [23], NIHR [29, 30], INVOLVE [16, 26, 31–33] and National Children’s Bureau (NCB) [17]. There was also one PIC related study on the INVOLVE research project database of family needs after Traumatic Brain Injury (TBI) [34]. This study described the impact of PPI to a research project development, but there was a lack of information about how the families had been identified and the recruitment process so we have excluded this study from this review.

**Table 2:** Characteristics of included studies

Study	Method & No. participants	Lay involvement & no. participants	Identification of participants	Outcomes
Tume et al. [35]	• Interviews: face to face (2) telephone (1) • Focus group (13)	• Former PIC patient (1) • Parents of former PIC pts (2). • NIHR YPAG (13)	• Posters/flyers in local Hospital • National charity • PIC National Audit Group (PICANet) • On line discussion forum • Approach of NIHR YPAG	'Insight' • Importance of national ventilator weaning study • Outcomes to be measured • Consent requirements • Identification of difficulty of recruiting PPI participants
Menzies et al. [36]. Abstract only	Focus group (6)	NIHR YPAG (6)	• Approach NIHR YPAG	'Project development' • Development of Participant Information Sheets (PIS) for a qualitative research study • Defining research protocol • Development of interview schedule
Menzies et al. [37]. Abstract only	Focus group (8)	Parents of children admitted to PIC (2008-2009) with Refractory Status Epilepticus (RSE) (8 parents representing 5 children)	• PIC admissions screened. Participants invited to attend focus group	'Insight' • Views on design of relevant and acceptable trial in context of RSE as an emergency situation. Views included: decision making, deferred consent, treatment failure, equity of access to treatments
Agrawal et al. [38]. Abstract only	Telephone interviews (12)	Parents of children admitted to PIC (2006–2007) with RSE (12)	• PIC admissions screened. Participants contacted by phone	'Insight' • Parent’s beliefs and attitudes towards clinical trials in RSE including: emergency situations and deferred consent, blinding, availability of different treatments. • Concluded further exploration though focus group required.

### Participants

Two of the papers made efforts to include children and young people within PPI activity [35, 36]. However, the majority of participants were representatives from the National Institute Health Research (NIHR) Children and Young Persons’ Advisory Groups (NIHR YPAG), none of whom had direct experience of PIC. Only one study directly engaged one child who had experience of PIC [35]. Three studies engaged with parents of children who had been patients on PIC [35, 37, 38]. Methods of identifying participants included: screening PIC admissions with an invitation by post to participate in PPI activity [37, 38], approach of a PIC associated charity (0 parents), use of an existing database of parents with experience of PIC (one parent) and an online discussion forum (0 parents) [35]. Despite using a variety of methods to engage with parents and families with PIC experience, Tume et al. [35] comment that recruitment to PPI was extremely challenging.

### Methods

Three of the papers used focus group discussion for the consultation process [35–37]. Both Menzies et al. [36] and Tume et al. [35] consulted with NIHR YPAG; an established group offering PPI support to all paediatric studies [29] and one study involved the set-up of a focus group specifically for the purpose of consultation [37]. Two studies conducted telephone interviews [35, 38] and one utilised face to face interviews [35].

### Reported outcome

Three studies specifically reviewed acceptability of research to parents and children; one in relation to a specific research project [35] and two in relation to potential future research [37, 38]. One study [36] engaged in ‘project development’, collaborating with participants to identify stakeholders, the development of an interview schedule and methods of engagement. All of the studies referred to PPI in positive terms but none of the studies provided an objective measurement of the impact of PPI.

### Quality

Three of the papers were reported in abstract only which made assessment of quality difficult [36–38]. The three abstracts did not feature a clear definition of PPI, focused on the results gained through consultation without comment or discussion about the PPI process and were therefore all rated as low quality. All four papers described a consultation approach to PPI and appeared to adopt a ‘once only’ approach, rather than involvement at multiple levels of the project. Tume et al [35] rated higher on the quality of reporting for the aims, background and methods but it was unclear about the contribution of PPI to the ongoing project development.

## Discussion

### Engagement with children and young people

There has been a shift in recent years towards recognising children as social actors with a unique perspective and insight [39], however within PIC research we found only one account of consultation with children with PIC experience. There are varying suggestions about the age at which is appropriate to engage with children in PPI. Guidance documents suggest involving those aged 12 years and older [40] and 13 years and upwards [17]. Involving very young children may not be appropriate as they can have difficulty communicating opinions [41] or comprehending abstract features of research such as risks [42]. However PIC service users are often far younger than these suggestions, with 47 % under the age of one year and within our institution less than 14 % are over 8 years of age (unpublished data). This could help to explain the finding that there are so few former PIC patients directly involved in PPI activity within the published literature [35]. However, the National Children’s Bureau [39] states there is no lower age limit at which children can participate in research, assuming the methodology is appropriate to the age group in question. Perhaps what is required is a change in mind set to adjust the methodology and research tools to those appropriate to our service users. This will improve accessibility and inclusion and ensure that we do not systematically exclude the majority of our patients from potential involvement. Future researchers need to consider how to engage with very young children, paying specific attention to the language, contribution to research, range of methods, involvement of parents and duration of activities [39]. Currently involvement of children in PIC research design has been predominantly through NIHR YPAG, with no experience of the PIC setting. We would like to see more efforts to engage with children who have insight into the experience of a PIC admission and recovery process.

There are challenges associated with approaching children due to the complex nature of PIC [43]. Patients on PICU are often acutely unwell and experiencing multiple organ dysfunction [44] with high numbers requiring invasive ventilation (up to 87 %). They are also likely to be receiving sedation and analgesia, affecting their comprehension and memory [45], and have barriers to reading and mobility such as intravascular catheters and splints as well as pain and fatigue [46]. The PIC physical environment is often space confined or poorly ergonomically designed, noisy and lacking privacy and the acuity means there is a frequent need for staff interventions [47, 48]. It is therefore extremely difficult to communicate with a child effectively about PPI whilst they are a patient on PIC. Length of stay is also relatively short for many patients, with over 80 % of patients on PIC for less than 7 days. Eighty percent of children have a single admission to PIC, which could limit their recollection of their time. Following up these patients can also be difficult as around 18 % of patients are discharged to another hospital, a hospice or even their home. For those discharged to inpatient wards there are difficult challenges about timing such as protected meal times, ward rounds, procedures, and privacy and personal care requirements. In addition it is important that parents are present and aware of the approach and potential future involvement [39] and also in the appropriate receptive state [31]. Children with life-threatening illnesses require a robust evidence base for both physical and psychosocial aspects of care [28]. However there is evidence that RECs may deny studies approval when they are concerned about vulnerable or distressed participants [49]. Researchers must reassure RECs that services users have been involved in the research process to reduce the apprehension with which many REC members view the involvement of children as research participants [23]. Ultimately this could involve initiating discussions whilst the child is on PIC and considering ways of involving them even whilst they are an inpatient.

### Engagement with parents and carers

Nuffield Council on Bioethics [23] states that partnership with parents who are in a similar situation to potential participants will help to ensure that important aspects of the research question have been considered. The concept of ‘similar situation’ does pose some challenges to researchers within PIC. Children in the PIC represent a wide age range and a broad case mix of underlying diseases and diagnosis [50]. Admissions cover a variety of specialities and there can be huge variation in the needs of families. Can parents of children who have been admitted following routine, elective procedures comment on research into trials of an emergency intervention? Do researchers need representation from different specialities to understand the stresses of admission to the PIC environment? The ideal situation would be to have sufficient respondents to consult with to ensure that different scenarios and different topic areas can be addressed and also PPI participants can contribute where they feel they are best placed or able to contribute. However, there is currently no local or national support group for parents whose child has had a PIC admission or ‘PIC parent group’. This increases the difficulty of accessing parents with the appropriate insight. Three of the publications identified consulted with parents, however there were specific references to PPI being challenging to recruit to, even when the study is of specific relevance to their child. Both Agrawal et al. [38] and Menzies et al. [37] conducted PPI activities within the emergency management of Refractory Status Epilepticus (RSE). Of 56 families approached for participation there were 47 non-responders and only representatives from five families went on to be involved in a focus group [37]. Even when the method was a telephone interview, only 35 % (12/34) of parents approached agreed to participate [38]. There is insufficient evidence to conclude how best to conduct PPI with parents and carers, particularly in research with critically unwell children.

There are a growing number of technology dependent, complex care patients who experience multiple admissions to hospital and to PIC [51]. They therefore have valuable insight into the PIC environment however the complex needs of their children may hinder their involvement. In addition, many parents of children admitted to PICU suffer from acute and post-traumatic stress disorder and this can last for months after discharge [52–55]. Affected individuals often avoid stress-provoking stimuli or reminders of traumatic events or experiences [56, 57] and may not wish to return to a hospital or clinical environment. However this does not mean excluding parents of children who have had prolonged admissions or even bereaved families, as there is evidence that people who have been bereaved have reported participation within research as a positive experience [58, 59]. Research with bereaved parents can be undertaken safely and ethically provided there is attention to the timing, approach and care of participants [59]. We believe this could extend to offering them the opportunity to participate with PPI activity appropriate to their knowledge and experience, although we did not find any published accounts to support this.

### Methods of engagement

The published articles describe a variety of approaches to identifying parents of children who have experienced an admission to PIC to PPI activities. Tume et al. [35] utilised wide inclusion criteria and a variety of recruitment methods for PPI participants locally and nationally. Agrawal et al. [38] and Menzies et al. [37] conversely screened and recruited for PPI participants from a specific population of PIC service users at one site. All studies struggled with recruitment to PPI. Conducting work as ‘standalone’ projects requires significant time to set up and facilitate and is not always productive [35]. Both Tume et al. [35] and Menzies et al. [36] accessed support from NIHR YPAG, who have a remit to help the development of clinical research [60]. Successful PPI is reported to occur when roles within PPI are agreed, training and support have been provided to enable consumers to be involved and consumers are kept involved and informed about the progress of research [4, 61]. However, this resource is not available nationally and the group have other competing objectives and priorities which can mean problems with achieving timely access. This model of sustained input requires significant resources to maintain and researchers are therefore advised that wherever possible to utilise existing PPI resources [46]. However, there are limited options available to consult with parents as there are no existing parent groups in the manner of the NIHR YPAG. There are databases of individuals willing to get involved in health and social care research and these are a key resource for researchers looking for PPI support in other areas of health care [62, 63]. Tume et al. [35] approached the PICANet Parent and Family Group and this method yielded one participant. However, this group has limited lay representation and does not have an established database of contacts for researchers to liaise with. There are therefore limited resources currently available for researchers wishing to conduct PPI with parents. One suggestion would be to develop a local database of PIC former service users and their parents or carers. We would suggest screening on discharge for children and parents who would be interested in being contacted for future consultation. Researchers need to consult colleagues who are familiar with the family to determine children with particular needs [39], as well as parents and carers as gatekeepers [17, 41]. VIPER [64] advise asking directly about access requirements and what someone needs to take part, rather than what condition they have. This will enable researchers to identify the individual child’s needs, how best to include them and wherever possible additional resources and support staff can be put in place [31, 39, 40].

Inviting known parents to participate in PPI activities is one solution clinicians have used. A recent survey showed 50 % of PPI representatives involved in research projects were patients or service users known to the clinician [65]. However there are challenges of managing patients’ expectations and the issues of raising aspects which imply criticism of their clinicians or service [66]. One suggestion to avoid this would be to involve service users only after their PIC admission to avoid role conflicts. However, as many patients will have numerous admissions over their lifetime this does not remove the issue of an ongoing care relationship with patients and their families. There would be value to a national database for the PIC community, however there are many issues about how this would be funded, organised and sustained which we do not currently have the answers to.

### Siblings and other relatives

PIC admission affects not only the individual child and parents but also the wider family unit [67]. There is evidence that siblings can have a good understanding of the PIC environment and their sibling's illness [68] and this could extend to becoming involved in PPI activities within PIC. Although none of the studies specifically referred to engaging with siblings of children there are accounts of members of the NIHR YPAG becoming involved due to their experiences as a carer of a family member, often a sibling [69]. Recruitment of siblings into PPI activities would require parents’ consent and this could be challenging to initiate whilst on PIC. It may also prove difficult for researchers to assess suitability and appropriateness of approach because PIC visiting might be restricted by school or distance from home. Siblings may not feel able to talk confidently about the PIC context as visiting can be restricted by care requirements, parental anxieties and avoidance of over-stimulation of the critically ill child [67, 70]. There is also the potential that other members of the family such as grandparents, aunts or uncles would also be able to participate in future PPI consultation. Individual family dynamics can be difficult to negotiate and this would again require careful assessment to determine knowledge, experience, agenda and expectations.

### Charities and support services

If there are no existing resources that can be accessed or there are issues about eligibility, support could be sought from an alternative source such as a charity or a support service [71]. They may be willing to contact members to assist with a specific project if the study is relevant to them and their service users [71, 72]. Tume et al. [35] adopted this approach, although ultimately this did not yield any participants. It is also possible to conduct PPI activities within the school setting [46], although we did not find reference to this utilised by PIC researchers in the published literature. There are a number of benefits of involving schools in research, including reducing the risk of burdening those undergoing treatment of care and supporting access to children and young people from different communities, age groups and backgrounds. Schools can provide a meeting space that is generally neutral [46]. The NIHR support recommendations to work with the education sector to promote clinical research in schools [30] and there are even cases of researchers facilitating YPAGs within the school setting to advise on research design and to promote the perspective of a lay paediatric service user [73]. This could be an interesting avenue to explore in future PIC PPI, particularly if the study required a PIC naïve perspective.

### Social media

Social media offers multiple new avenues to engage with service users. INVOLVE offer a website that allows researchers to advertise for people to become involved in specific research projects (www.peopleinresearch.org/) [32] which could be of use for future PIC studies. There are also examples of researchers in a variety of areas using social media to recruit patient and public involvement through Facebook, Twitter, and blogging. Tume et al. [35] describe the use of an online discussion forum to advertise for interested parents. Although this was not successful in this instance, it is possible this will develop as the use of social media increases. Although there are benefits for both researchers and participants to the traditional face to face engagement, the PPI literature suggests communicating with children and young people in a variety of ways; including online, text, phone and post [16]. There is no one ‘best’ method for conducting consultation or research [74]. Using methods such as conference calling, social media and virtual forums [30, 31] might allow people to participate who might otherwise not have the time, energy or transportation means. Being flexible and considering different methods of engagement will assist with inclusivity and access for a wider range of service users. These methods were not widely reflected within the published PPI literature in the PIC context, but future researchers would be wise to explore how technology and social media may assist.

As researchers in the PIC environment we had experienced difficulties with meeting the requirements of RECs and funding bodies for good quality PPI. We conducted this review to examine the current PPI evidence base and try to consolidate what is currently known about consumer involvement in PIC research design. The published articles suggest there is benefit to research design through enhanced insight into service users perspectives [35, 37, 38] and the development of the research protocol and research instruments [36]. However, none of the projects provide an indication of how this enhanced understanding contributed to future project development. This is a common phenomenon amongst PPI literature. In a review of the public involvement literature in research, only 22 % of papers included evidence of the impact within the article [75]. Future PPI work in the PIC context needs to provide further information to aid researchers to establish the difference PPI makes as well as the best ways to conduct it [76]. All of the papers included within the review were conducted as ‘one off’ consultations. We found one example within the INVOLVE research project library of a project with multi-stage PPI and examples of impact [34] however there was a lack of detail about who the families were, how they had been identified as well as the method and duration of consultation. Future research needs to improve on the quality of reporting on all of these aspects to assist with evaluation.

There are a number of generic guidance documents to advise researchers on the conduct of PPI with children and young people [16, 26, 31–33, 77]. However, these do not take into account the specific considerations for PIC. We have attempted to summarise recommendations for researchers wanting to engage with the public about the design of PIC research studies. Please see Table 3 below.

**Table 3:** Recommendations for future PPI in PIC context

• Use resources available to researchers concerning PPI and service users, including existing PPI groups where available [26, 16, 17, 31–33, 40, 46, 84].	
• Develop a local PIC maintained database of former patients and parents. Consider approaching former patients and parents of children.	
• Consult with clinicians and families to identify children with specific needs or vulnerabilities and discuss ways to ensure inclusivity [39].	
• Follow up potential participants on the ward but consider approach on PIC if it is appropriate for the individual, particularly where multiple admissions are likely.	
• Consider engaging with siblings and other relatives/carers of the child who has been admitted to PIC, but ensure sensitivity with parents as gatekeepers [17].	
• Consider how to engage with bereaved families or families with complex situations. Liaise with family support services to determine appropriateness and timing of approach. If become aware of issues of PTSD ensure that parents/carers are assisted to access services and support [55].	
• Use a variety of methods to advertise for PPI volunteers, with application forms with contact details to collate interested parties, including paper forms, flyers, posters and information on websites.	
• When meetings are face to face ensure the setting and time are convenient and large enough to accommodate all attendees needs [41].	
• Provide PIC naive PPI participants with training about context of PIC and plan visits so they can see the environment directly.	
• Provide research naive participants with training on research methods and concepts [61].	
• Consider PPI within school setting particularly for the perspective of a PIC naïve population [30].	
• Consider approaching charities or specialist support services to find appropriate PPI representatives [71, 72].	
• Utilise a variety of communication methods including social media, texting, Smartphone apps and newsletters [31]. Keep in touch regularly with updates [16].	
• Use a range of different methods of conducting PPI to facilitate participation from a wide group of people such as video conferencing, use of surveys and use of virtual forums [64].	
• Consider a specific request for participants through use of INVOLVE resource (www.peopleinresearch.org/) [32].	

## Limitations of the review

One challenge to the identification of PPI literature in the PIC setting was the difficulty associated with terminology. Although PPI is a growing phenomenon there is no MeSH term [25, 78] and we found the searches to be high yielding but non-specific. We have used search terms recognised by PPI contributors but we acknowledge that the search may have lacked sensitivity. PPI has often been poorly reported which can make it difficult to determine the nature of the activity and the impact of the intervention [27]. We have included papers which specifically referred to consultation at the project development stage rather than where participants have provided their insight as research participants; however this was difficult to differentiate at times. We have tried to be inclusive, including three abstracts published in abstract form only. None of these abstracts have yet to be published as a full account of PPI activity (confirmed with authors). These were rated as low quality using the GRIPP checklist and all four studies failed to provide any examples of how the conduct of PPI had impacted on the project development in a measurable way. PPI is reported to enhance the quality and appropriateness of research, particularly if service users are involved throughout the study [3]. However, all of the published papers described PPI in terms of an isolated event with no ongoing involvement at any future stages. Hopefully this situation will improve with future emphasis on the benefits of more collaborative working.

## Conclusions

Critically ill children and their families should have the opportunity to participate in translational or clinical research and have access to interventions supported by a firm evidence base [79, 50, 80]. However, designing and conducting research within the PIC context is extremely challenging [81]. PPI offers the opportunity for researchers to engage meaningfully with children and their families and conduct a full assessment of the risks and benefits of carrying out research. However, there are few published accounts of PPI with parents of children admitted to PIC and only one account of engagement with former PIC patients. The lack of engagement with children who have been patients on PIC needs to be addressed, with the consideration of multi-method, flexible approaches which can be tailored to the age and needs of those involved [39].

There have been varied approaches to the identification and recruitment of participants to PPI and a variety of methods to conduct PPI. However, the lack of published detail about the impact of involvement means it is difficult to draw conclusions about why, how and when involvement brings the greatest benefits. This is essential to understand what type of involvement is most likely to bring added value [8]. All of the papers were positive about the effects of PPI on their understanding and development of a research project, however similar to much of the wider PPI literature there was no measurement of impact [3, 75, 82]. Future PPI reports need to provide measures of impact, both positive and negative, to develop a more comprehensive understanding of what PPI works, for whom and in what circumstances [82]. From this the evidence base will grow and the PIC community as a whole can learn how to optimise PPI [66].
